# The Role of a Pathological Interaction between β-amyloid and Mitochondria in the Occurrence and Development of Alzheimer’s Disease

**DOI:** 10.32607/actanaturae.11723

**Published:** 2022

**Authors:** N. S. Nikolaeva, E. Yu. Yandulova, Yu. R. Aleksandrova, A. S. Starikov, M. E. Neganova

**Affiliations:** Federal State Budgetary Institution of Science Institute of Physiologically Active Compounds of the Russian Academy of Sciences, Chernogolovka, 142432 Russia

**Keywords:** Alzheimer’s disease, beta-amyloid, mitochondria, MAM, mitophagy

## Abstract

Alzheimer’s disease (AD) is one of the most common neurodegenerative diseases in existence. It is characterized by an impaired cognitive function that is due to a progressive loss of neurons in the brain. Extracellular β-amyloid (Aβ) plaques are the main pathological features of the disease. In addition to abnormal protein aggregation, increased mitochondrial fragmentation, altered expression of the genes involved in mitochondrial biogenesis, disruptions in the ER–mitochondria interaction, and mitophagy are observed. Reactive oxygen species are known to affect Aβ expression and aggregation. In turn, oligomeric and aggregated Aβ cause mitochondrial disorders. In this review, we summarize available knowledge about the pathological effects of Aβ on mitochondria and the potential molecular targets associated with proteinopathy and mitochondrial dysfunction for the pharmacological treatment of Alzheimer’s disease.

## INTRODUCTION

Neurodegenerative diseases are disorders characterized by the progressive death of the neurons associated with the deposition of proteins, with altered physicochemical properties and severe cognitive impairment. It is estimated that the number of people with dementia will increase to 131.5 million worldwide by 2050 [1]. Alzheimer’s disease (AD) is the most common form of neurodegenerative diseases; it develops mainly in people over 65 years of age [2]. The key pathomorphological features of AD include deposition and accumulation of abnormally folded β-amyloid (Aβ) peptide and truncated/hyperphosphorylated tau proteins [3, 4]. The cause behind AD development remains controversial and not completely understood. Various hypotheses of AD pathogenesis have been proposed, the most common of which are the hypotheses of the amyloid [5, 6] and mitochondrial cascades [7]. The cholinergic [8] and tau [9] hypotheses, the theory of oxidative stress (OS) [10, 11], hypotheses of calcium homeostasis [12] and neuroinflammation [13], the neurovascular hypothesis [14], hypotheses based on metals with a variable oxidation state [15] and viral origin [16] were also proposed. To date, there is no drug that can prevent AD from developing. Four drugs are used in clinical practice: three cholinesterase inhibitors (galantamine, rivastigmine, and donepezil) and memantine (a non-competitive NMDA receptor antagonist). However, these drugs have symptomatic effects only. Therefore, an intensive search for new potential drugs based on the data postulated in modern hypotheses of AD pathogenesis is currently under way.

There are sporadic (found in most cases) and familial (inherited in an autosomal dominant manner; has an early onset) forms of AD. The familial AD results from mutations in the genes encoding the β-amyloid precursor protein (APP; located on the chromosome 21) [17], presenilin 1 (PSEN1, located on the chromosome 14) [18], and presenilin 2 (PSEN2, located on the chromosome 1) [19]. The presence of one or more mutations in these genes leads to impaired APP cleavage, resulting in an increased ratio of Aβ1-42/Aβ1-40 peptides [20, 21], which, in turn, causes deposition of fibrillar Aβ and an early onset of the disease [22, 23]. The sporadic AD, which has a late onset, is a multifactorial pathological condition resulting from allelic variation in apolipoprotein E (APOE), vascular pathologies, immune system defects, mitochondrial dysfunction, and dyshomeostasis of metals with a variable oxidation state [24].

One of the important pathogenic mechanisms of AD is the malfunction of the main energy-generating organelle of the cell: the mitochondrion. Mitochondria are two-membrane organelles that undergo fission and fusion cycles, leading to changes in the organelle dynamics, morphology, and functions [25]. Mitochondrial dysfunction plays an important role in the pathology of neurodegenerative diseases [26 , 27, 28]. The mitochondrial fission/fusion balance, their biogenesis, ubiquitin–proteasome pathways, as well as mitophagy and autophagy signaling proteins, determine the physiological state of newly formed mitochondria. Aβ and the hyperphosphorylated tau protein are involved in the oxidative damage inflicted on mitochondrial membranes and mtDNA, which ultimately leads to an imbalance in mitochondrial dynamics [29]. Aβ-induced OS alters mitochondrial fusion/fission, worsening the state of organelles and increasing the level of reactive oxygen species (ROS), molecular markers of OS. This, in turn, leads to the accumulation of pathological Aβ. The main routes through which Aβ enters mitochondria are the mitochondria-associated endoplasmic reticulum membrane (MAM) and the complex of outer and inner membrane translocases (TOM–TIM) [30, 31].

In our review, the main pathways of mitochondria– Aβ interaction are associated with the Aβ intake, excretion, and effect on the various mitochondrial functions. These pathways can serve as potential targets for neuroprotective drugs that can prevent both Aβ deposition and mitochondrial dysfunction, as well as delay AD progression.

## PATHWAYS OF Aβ FORMATION FROM THE AMYLOID PRECURSOR PROTEIN

**Fig. 1:**
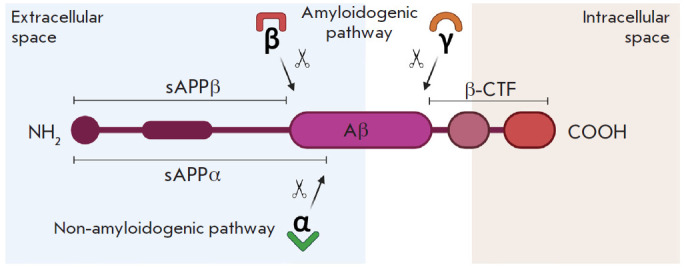
Simplified representation of the APP structure and cleavage. APP undergoes sequential proteolysis by β-secretase (β), α-secretase (α), and γ-secretase (γ) to release Aβ from the neuronal plasma membrane. sAPPα – soluble alpha fragment of APP; sAPPβ – soluble beta fragment of APP; a CTF-β fragment (C99, membrane-associated)

The Aβ peptide forms through sequential cleavage of APP by α-/β- and γ-secretases [32]. APP is a type I membrane protein (110–130 kDa) containing a large extracellular glycosylated N-terminal domain and a shorter cytoplasmic C-terminal region located towards the intracellular space. APP is synthesized in the ER and then transported to the Golgi complex, where it completes its maturation. Its mature form is then transported to the plasma membrane [33]. There are two pathways of APP cleavage: non-amyloidogenic, which prevents Aβ deposition, and amyloidogenic, which results in Aβ formation (Fig. 1).

In the non-amyloidogenic pathway, the first cleavage of APP is catalyzed by α-secretase, the enzyme that belongs to the disintegrin family, and ADAM metalloproteases (Disintegrin and the metalloproteinase domain-containing protein; ADAM10 [EC 3.4.24.81] and ADAM17 [EC 3.4.24.86] in neurons). The plasma membrane and the trans-Golgi network are considered to be the main sites of APP cleavage by α-secretase [34]. The α-secretase enzyme cleaves APP in the Aβ sequence between the amino acids 16 and 17 to form a small membrane-anchored 83-amino-acid C-terminal fragment of APP (α-CTF, C83) and soluble APP-α (sAPPα) [35]. sAPPα is known for its numerous neuroprotective functions; in particular, it counteracts the toxic effects of Aβ [36, 37]. Next, α-CTF is cleaved by γ-secretase to generate the hydrophobic P3 peptide (3 kDa) and intracellular domain of the amyloid precursor protein (AICD) [38]. The functional γ-secretase complex includes the following proteins: either presenilin 1 (PS-1) or presenilin 2 (PS-2), which belong to the catalytic domain; nicastrin, which serves as a substrate receptor [39]; presenilin-enhancer-1 (Pen-1, or aph-1; anterior pharynx-defective 1) and presenilin-enhancer-2 (Pen-2) [40]. Aph-1 and Pen-2 act similar to the transmembrane aspartate protease, playing an important role in the Aβ_1-40_/Aβ_1-42_ ratio [41].

The amyloidogenic pathway begins with N-terminal cleavage of APP by β-secretase (BACE1; β-Site APP-cleaving enzyme 1 [EC 3.4.23.46]) [42] resulting in the formation of soluble sAPPβ and the β-C-terminal fragment (β-CTF; 99-amino-acid C-terminal fragment of APP; C99). Next, the γ-secretase complex cleaves β-CTF to generate Aβ (4 kDa) and AICD [35]. The Aβ_1–42_ form is more toxic than Aβ_1–40_ due to its higher tendency to form aggregates [43]. Aβ1–42 activates signaling pathways that lead to synaptic and mitochondrial dysfunction, disruption of Ca^2+^ homeostasis, onset of OS, and, ultimately, neuronal apoptosis [44]. Accumulation of Aβ and C99 stimulates neuroinflammation in a mouse model of AD [45, 46]. Aβ is localized in extracellular and intracellular compartments, including endosomes, lysosomes, and the mitochondrial membrane [47, 48].

Thus, Aβ is formed via the pathological amyloidogenic pathway, either in the case of mutations in the genes encoding γ-secretase complex proteins or in disrupted expression of the α- and β-secretase enzymes, resulting in the formation of a longer Aβ peptide capable of aggregation.

## PATHWAYS OF Aβ INTAKE BY MITOCHONDRIA AND ITS EFFECT ON MITOCHONDRIAL TRANSPORT

**Fig. 2:**
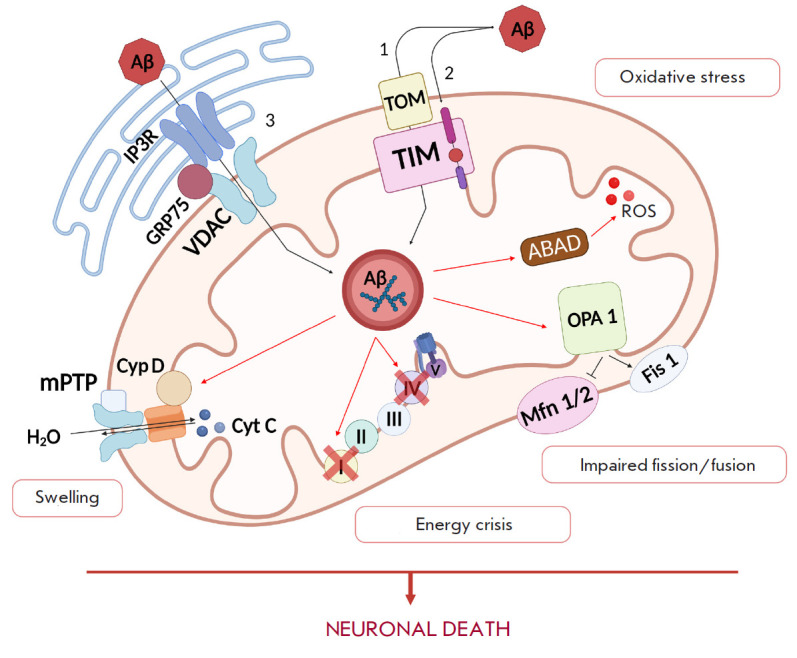
Schematic representation of the pathways of β-amyloid (Aβ) entry into mitochondria and its pathological effects on these organelles. The first pathway is via the TOM–TIM complex. This pathway has two options: (1) Aβ enters the mitochondrial matrix; (2) Aβ binds to TIM, thus disrupting the import of important mitochondrial proteins. The second pathway is performed through the endoplasmic reticulum (ER)–mitochondria contact sites MAM (3). The formation of Aβ in MAM increases Ca^2+^ entry into mitochondria from the ER through the IP3R–GRP75–VDAC channel. The Aβ–alcohol dehydrogenase (ABAD) complex induces ROS formation. Aβ inhibits fusion proteins (OPA1 and Mfn1\2) and activates the fission protein (Fis1), resulting in the formation of abnormal mitochondria. Aβ binding to cyclophilin D (CypD) leads to the opening of the mitochondrial permeability transition pore (mPTP). Aβ accumulation in mitochondria disrupts the ETC function, which leads to the formation of ROS and further death of neurons

Normal functioning of mitochondria requires a large number of proteins, the majority (about 99%) of which are synthesized in cytosolic ribosomes [49] and imported post-translationally into various subcompartments of organelles. To date, several pathways for Aβ (and many other mitochondrial proteins) import directly into mitochondria are known: via translocases of the outer (TOM) and inner (TIM) membranes and through MAM sites (Fig. 2). In addition, Aβ can form directly in mitochondria as a result of APP cleavage by γ-secretase [50, 51].

The TOM complex consists of the main protein TOM40 and adaptor TOM70, TOM22, and TOM20 (large) and the TOM7, TOM6, and TOM5 (small) proteins. Large TOMs are involved in protein recognition, while the small ones participate in pore formation [52]. Protein import from the inner membrane requires the recruitment of the TIM complexes (TIM23 and TIM22) [53]. A decrease in Aβ_1-40_ and Aβ_1-42_ import in the presence of antibodies to the mitochondrial receptors TOM20 and TOM70 or to the common mitochondrial import pore of the outer membrane, TOM40, confirms that Aβ enters mitochondria through the TOM–TIM complex [50]. The Aβ peptide does not affect the structure of translocase systems but significantly hinders mitochondrial preprotein transport via the extramitochondrial coaggregation mechanism [54].

Aβ is translocated from the ER membrane into mitochondria through the contact sites between these organelles called MAMs [55], which have the characteristics of a lipid raft and are rich in cholesterol and sphingomyelin [56]. The physiological functions of MAM include the regulation of phospholipid and Ca^2+^ homeostasis, mitochondrial fusion/fission, apoptosis, autophagy, and cholesterol esterification [57, 58]. MAM is enriched in sarco/ER Ca^2+^ ATPase (SERCA) [59] as well as the sigma-1 (Sig-1R) [60] and inositol-1,4,5- trisphosphate receptors (IP3R) [61]. The ER and mitochondria interact through mitofusin-2 (Mfn-2) and cytosolic chaperone Grp75 (a member of the heat shock protein 70 family), which is associated with IP3R on the ER membrane and with voltage-dependent anion-selective channel 1 (VDAC1) on the mitochondrial membrane. VDAC1 is a multifunctional protein expressed in mitochondria and other cell compartments, including the plasma membrane, and a key regulator of Ca^2+^ homeostasis, OS, and apoptosis [62]. The IP3R–GRP75–VDAC complex regulates Ca^2+^ transfer from the ER to mitochondria [63]. MAM functions are disturbed in cell pathologies, which leads to increased ER stress (accumulation of aberrant unfolded/misfolded proteins in the ER lumen, followed by their aggregation) [64], and disruption of Ca^2+^ homeostasis. Hedskog et al. demonstrated the ability of nanomolar concentrations of the Aβ peptide to increase both the expression of IP3Rs and VDAC and the number of ER–mitochondria contacts and, thereby, increase Ca^2+^ concentration in organelles [65]. The interaction between VDAC1 and Aβ leads to mitochondrial pore dysfunction. This disrupts the transport of mitochondrial proteins and metabolites of up to 150 kDa (ADP and inorganic phosphate), which are necessary for the completion of oxidative phosphorylation and ATP synthesis. Abnormal transport of proteins and metabolites leads to impaired oxidative phosphorylation and mitochondrial dysfunction [66]. VDAC1 overexpression in the human cerebral cortex correlates with the stages of AD; this is also observed in mice transgenic for the APP gene and Aβ-exposed neuroblastoma cells. A decrease in VDAC1 expression is accompanied by a drop in the levels of APP and BACE1 mRNA [62].

Data on a possible formation of Aβ directly in MAM has been published [67]. The presence of presenilins and the C99 fragment, which is cleaved by γ-secretase [69], in MAM [68] may explain the mito chondrial localization of Aβ [50]. In addition, MAM is a lipid raft-like domain [70], while APP cleavage via the amyloidogenic pathway depends on the lipid raft [71, 72]. A change in γ-secretase activity leads to the accumulation of the C99 fragment in MAM, inducing esterification of cholesterol, hydrolysis of sphingolipids, and mitochondrial dysfunction [73]. It has been suggested that the synergetic effect of ceramide, a product of sphingomyelin hydrolysis, and Aβ can cause neuronal death in AD [74]. Mutations in PSEN1, PSEN2, and APP upregulate MAM function and significantly increase the ER – mitochondria interaction [75].

Takuma et al. showed that the receptor for advanced glycation endproducts (RAGE, type I transmembrane protein) also facilitates Aβ1-40 translocation from the extracellular to the intracellular space, which can be one of the mechanisms of Aβ import into mitochondria [76]. Aβ accumulation in the brain leads to RAGE overexpression in the affected vessels, neurons, and microglia [77], which, in turn, induces ROS production, mainly due to the activity of NADPH oxidases [78].

Aβ accumulates on the inner mitochondrial membrane [79], hindering the import of the precursor proteins required for mitochondrial biogenesis [54]. Aβ also interacts with cytochrome c oxidase, F1α ATP synthase, and the subunits of the electron transport chain, while inhibiting the activity of the complexes [80]. For instance, 24 proteins were found to be dysregulated in transgenic pR5/AβPP/PS2 mice; one-third of these proteins are mitochondrial proteins associated mainly with oxidative phosphorylation system (OXPHOS) complexes I and IV [81]. It is noteworthy that complex IV dysregulation depends on the level and degree of Aβ activity. In addition, Aβ accumulation in mitochondria correlates with manifestations of early synaptic deficit in a mouse model of AD [82, 83].

The Aβ was shown to enter mitochondria through translocases of the mitochondrial membrane and at the ER–mitochondria contact points. Moreover, Aβ is synthesized directly in mitochondria as a result of APP cleavage by γ-secretase localized in them, which leads to mitochondrial transport dysfunction.

## EFFECT OF Aβ ON MITOCHONDRIAL DYNAMICS AND BIOGENESIS

Mitochondrial biogenesis is a complex process involving the nuclear and mitochondrial genomes and resulting in an increased number of mitochondria in response to enhanced energy demand. Peroxisome proliferator-activated receptor-γ 1α coactivator (PGC-1α) is a master regulator of mitochondrial biogenesis, energy metabolism, and respiration through interactions with various transcription factors, including nuclear respiratory factors 1 (NRF-1) and 2 (NRF-2) [84]. Qin et al. were the first to show a decrease in PGC-1α expression in AD patients and a transgenic mouse model of AD [85]. Administration of PGC-1α in the hippocampus and the cerebral cortex of transgenic APP23 mice decreased the level of Aβ deposits owing to BACE1 downregulation and helped to preserve most neurons [86]. Exogenous PGC-1α expression in neuroblastoma N2a cells suppresses BACE1 transcription, which, in turn, reduces the level of secreted Aβ and increases the level of sAPPα [87]. PGC-1α activity is regulated by AMP-activated protein kinase (AMPK) and sirtuins (SIRTs). Aβ was found to cause overexpression of poly(ADP-ribose) polymerase 1 (PARP1 [EC 2.4.2.30]), which is accompanied by NAD^+^ depletion followed by a reduction of SIRT1 activity. Inhibition of PARP1 induces SIRT1 expression, leading to an increase in α-secretase expression, downregulation of BACE1, and a decrease in the Aβ level [88]. Small interfering RNAs (siRNAs), a group of small single-stranded non-coding RNAs involved in mitochondrial biogenesis and post-transcriptional regulation of mRNAs by inhibiting their translation and degradation, also affect SIRT function [89]. These non-coding RNAs are also involved in AD pathogenesis [90 , 91, 92, 93].

Mitophagy is a process in which damaged mitochondria are specifically taken up by autophagosomes and subjected to lysosomal degradation, which prevents the accumulation of dysfunctional mitochondria [94]. The main mitophagy pathway is ubiquitin-and receptor-mediated mitophagy; PTEN-induced kinase 1 (PINK1) and Parkin play an important role in this process. An abnormal increase in the number of autophagic vacuoles containing defective (aberrant) mitochondria with an altered activity of PINK1 [EC 2.7.11.1] and Parkin [EC 2.3.2.31] is observed in AD [95]. Aβ and hyperphosphorylated tau cause oxidative damage to mitochondria, resulting in a reduced level of these proteins [96, 97, 98]. This leads to a decrease in the number of completed mitophagy processes and contributes to an increase in the number of Aβ and tau aggregates. Vaillant-Beuchot et al. showed that, independent of Aβ, the C-terminal fragments of APP trigger excessive disorganization of mitochondrial cristae, enhance ROS generation, and reduce the mitophagy associated with insufficient fusion of mitochondria with lysosomes [99].

Not only changes in mitochondrial morphology, but also disrupted distribution of these organelles in the brain cells are observed in AD. Anterograde (kinesin-based) transport promotes the delivery of newly formed mitochondria to axons; retrograde (dynein-based) transport promotes the removal of damaged organelles and maintains a healthy level of their population [100]. Disruption of the transport system and the balance between healthy/damaged mitochondria can change the distribution of organelles, which, in turn, has a significant impact on the synaptic and neuronal functions [101]. Aβ reduces the expression of the anterograde KIF5A protein [102], while interaction between oligomeric Aβ and the dynein intermediate chain negatively affects dynein interaction with snapin (adaptor protein) [103]. Mutations in the PSEN1 impair axonal transport by activating glycogen synthase kinase-3β (GSK-3β), which phosphorylates the kinesin light chain and releases it from the sites of its incorporation into the membrane [104].

Mitochondrial transport is important for neuronal survival, given the need for a proper distribution of mitochondria in areas with a higher demand for ATP and calcium. In addition, mitochondria are organized into a dynamic network through the continuous cycles of fusion and fission necessary for mitochondrial homeostasis and adaptation to cellular needs [105, 106]. Fusion and fission of mitochondria are regulated by proteins of the dynamin family, which have GTPase activity. Mitochondrial fission involves the proteins Fis1 (mitochondrial fission protein 1) and Drp1 (dynamin-like protein 1, DLP1), while fusion is mediated by mitofusins (mitofusins Mfn1 and Mfn2 are involved in outer membrane fusion) and the protein encoded by OPA1 [107, 108]. The imbalance between mitochondrial fusion and fission has been confirmed in in vivo studies [109]. Overexpression of wild-type (APPwt) and mutant (APPswe) APP in M17 neuroblastoma cells and primary neurons leads to mitochondrial fragmentation and their perinuclear distribution through a decrease in the levels of the fusion proteins, in particular Drp1, OPA1, Mfn1, and Mfn2, and an increase in the level of mitochondrial Fis1. These effects are blocked by the BACE1 inhibitor, which indicates that Aβ affects mitochondrial fragmentation [110, 111].

Mitofusins, located on the outer mitochondrial membrane, are involved in fusion by forming homotypic and heterotypic interactions with the OPA1 protein of the inner mitochondrial membrane [112]. It has also been reported that Mfn2 is present in MAM; it regulates axonal transport [113] and modulates γ-secretase activity and Aβ formation [114].

Drp1 is a mitochondrial fission protein that is involved in cell fragmentation, phosphorylation, ubiquitination, and death [115, 116]. An interaction of oligomeric Aβ and hyperphosphorylated tau with Drp1 was uncovered in the brains of AD patients and transgenic mice [117]. ROS are formed during the interaction between Aβ and Drp1 and are further involved in mitochondrial fragmentation [118], followed by mitochondrial depletion in axons and dendrites, resulting in the loss of synapses [119]. At the same time, Aβ-induced OS and calcium entry into the cell lead to Drp1 phosphorylation, causing an increase in the activity of extracellular signal-regulated kinase (ERK) and Akt [20, 121].

Thus, pathological Aβ negatively affects many important mitochondrial functions, leading to a disruption of their biogenesis, transport system functioning, the balance between damaged and healthy mitochondria, and, as a result, changes the distribution of these organelles in neurons, which, in turn, affects the synaptic and neuronal function.

## Aβ CLEAVING ENZYMES

results in its abnormal deposition in the brain tissue [122, 123]. The main pathways underlying Aβ elimination include its clearance through the blood–brain barrier (BBB), enzymatic degradation, cellular uptake, and subsequent degradation [124, 125]. The main enzymes involved in the extracellular cleavage of Aβ include the following zinc metallopeptidases: neprilysin (NEP [EC 3.4.24.11]), insulin-degrading enzyme (IDE [EC 3.4.24.56]), endothelin-converting enzyme (ECE [EC 3.4.24.71]), and matrix metalloproteinase-9 (MMP-9 [EC 3.4.24.35]) [126, 127]. Peptidases PreP [128] and transthyretin, which are capable of excreting amyloid by a mechanism similar to NEP [129], also exhibit catalytic activity against Aβ. Another peptidase neurolysin (NLN [EC 3.4.24.16]), which is capable of degrading mitochondrial precursor proteins ( < 20 amino acid residues long) and longer mitochondrial peptides, has been found in the mammalian mitochondrial matrix. An in vitro analysis of peptide cleavage revealed an interaction between NLN and PreP during the degradation of long peptides; in particular, the hydrophobic fragment of Aβ_35-40_ [130].

IDE is an extracellular zinc metallopeptidase capable of regulating the plasma levels of insulin, as well as extracellular Aβ. IDE is localized mainly in the cell cytosol [131]. However, it is also found in mitochondria and endosomes [132]. IDE selectively interacts with Aβ monomers [133]. The activity of this enzyme is determined by the dynamic equilibrium between soluble Aβ monomers and its aggregates [134]. Decreased levels of IDE and angiotensin-converting enzyme (ACE [EC 3.4.15.1]) and an increased Aβ level (due to slower exogenous protein cleavage) are observed in transgenic CB2R-/-Aβ_1-42_ mice lacking the cannabinoid receptor type 2 (CB2R) compared to WT-Aβ_1-42_ mice [135]. NEP is a type II integral membrane protein located in the plasma membrane; a larger part of this protein, including the active site, is located in the extracellular space [136]. Evidence has been obtained that the NEP and IDE activities are regulated by cholesterol levels. The enzymes IDE and NEP are sensitive to the OS caused by high cholesterol levels. In addition, their activity is also associated with APOE. High NEP activity was noted in the brain of people carrying the ε2 allele of APOE, while patients with the ε4 allele have decreased levels of IDE and NEP [137]. The activity of IDE and NEP is also affected by protein kinases A and C (PKA and PKC), which regulate the direct (enzymatic) cleavage of APP, thus decreasing the Aβ level. An experiment in a primary culture of rat astrocytes demonstrated that PKA activation impedes Aβ degradation by reducing the level of NEP but not IDE, while PKC activation stimulates NEP release into the extracellular space and IDE overproduction in astrocyte cell membranes [138].

Mitochondrial peptidasome (PreP, or PITRM1) is a metallopeptidase 1 located in the mitochondrial matrix and involved in the cleavage of protein pre-sequences after their import into mitochondria. Accumulation of Aβ was detected in the brain of mice heterozygous for PITRM1 [139]. Recent studies have revealed the role of PreP in Aβ metabolism [140]. For instance, PreP cleaves Aβ_1-40_, Aβ_1-42_, Arctic Aβ (E22G), and the 53-amino-acid mitochondrial pre-sequence pF1β [141, 142]. A significant decrease in the proteolytic activity of PreP against both the Aβ and non-Aβ peptides in mitochondria of the brain of transgenic mAβPP and mAβPP/ABAD mice should be noted [143]. At the same time, overexpression and increased PreP activity contribute to a decrease in the mitochondrial Aβ level [140]. Increased PreP expression not only leads to a degradation of mitochondrial Aβ, but also affects the overall level of Aβ in the brain. A decrease in the PreP activity in brain mitochondria is associated with functional changes in it; for instance, it can be due to protein oxidation [26]. PreP inactivation in acidic conditions has been shown to be due to the oxidation of cysteine residues and subsequent oligomerization through the formation of intermolecular disulfide bonds [144]. Disruption of the PreP function in OS is confirmed by Teixeira et al., who revealed the concentration dependence of PreP activity inhibition by hydrogen peroxide [145]. Thus, one can assume that increased ROS generation resulting in the inhibition of PreP activity is due to Aβ accumulation in mitochondria [146].

In addition, the acidic environment in mitochondria prevents Aβ clearance owing to its rapid interaction with cyclophilin D (CypD) and/or Aβ-binding alcohol dehydrogenase (ABAD) [147]. ABAD is a mitochondrial protein that contributes to the toxic effect of Aβ in mitochondria of AD patients and in a mouse model of AD by increasing ROS production and decreasing ATP levels [148, 149]. The formation of the ABAD–Aβ complex disrupts the interaction between NAD^+^ and ABAD, which changes mitochondrial membrane permeability [150] and accelerates mitochondrial dysfunction [151]. CypD is an important part of mPTP: it is responsible for its opening [152]. The formation of CypD–Aβ complexes causes mPTP opening, which leads to matrix swelling and ROS generation [153]. This, in turn, results in a disruption of the outer membrane and nonspecific release of such intermembrane proteins as cytochrome c, endonuclease G, procaspase, and Smac/DIABLO into the cytosol, where they activate apoptosis [154, 155]. A decrease in CypD expression leads to the suppression of Aβ- related disorders, in particular Ca^2+^-dependent mitochondrial swelling, a decrease in the calcium uptake, and an impairment of the mitochondrial respiratory function [156].

Thus, the importance of regulating the performance of the enzymes cleaving Aβ in both extracellular and intracellular spaces, as well as the factors inhibiting their activity, in order to reduce the toxic effect of Aβ on neurons has been mentioned.

## POTENTIAL NEUROPROTECTOR AGENTS ACTING ON BOTH Aβ DEPOSITION AND MITOCHONDRIAL DYSFUNCTION

One of the most common undertakings in the search for potential drugs against AD is the synthesis of compounds that reduce deposits of Aβ and prevent its accumulation in the first place. However, as various studies have shown, action on only one target is not enough to obtain a promising neuroprotector. For this reason, we studied the interaction between Aβ and mitochondria, in an attempt to combine the Aβ- aggregation-modulatory and mitoprotective effects in one molecule. By combining and systematizing data on compounds that could work against AD and are currently under study, one can outline promising fields and possible modifications to a molecule for the synthesis of more effective compounds.

Taking into account the multifactorial nature of AD, in particular the relationship between Aβ, mitochondria, and OS, pharmacological correction of mitochondrial dysfunction with a simultaneous effect on Aβ formation, deposition, and excretion seems a promising direction. Some potential multitarget compounds acting on the pathological processes described above are presented in Table 1.

**Table 1:** Potential multitarget agents for the treatment of Alzheimer’s disease

Agent	Aβ-associated targets	Mitochondrial targets	Main effect	Ref.
Epigallocatechin-3-gallate (EGCG)	NEP; BACE1	ROS and NO	↓ Aβ deposition; ↓ OS; ↑ learning and memory	[158] [160] [193]
Kai-Xin-San	NEP	LP; SOD, GPx, and CAT	↓ Aβ level; ↑ learning and memory; ↑ antioxidant system	[163, 164]
Curcumin	Aβ fibrils and oligomers; BACE1	ROS; SOD and GSH	prevents Aβ deposition; ↑ antioxidant system; ↓ OS	[178, 179] [216]
Silibinin	APP and BACE genes; NEP	LP; CAT, SOD, NO, and GSH	↑ antioxidant system; improves memory in animals	[183 , 184, 185, 186, 187]
Quercetin	APP, BACE, APH1 and PSEN1; ADAM10 and ADAM17	ROS, MDA, GPx, and SOD ↓ mitochondrial dysfunction;	↓ Aβ level	[190 , 191, 192, 193, 194]
Baicalein	Aβ; stimulates neurogenesis	OS	↓ neuronal death; improves memory in mice	[197, 198]
Berberin	BACE1	ROS; SOD	↓ Aβ level; improves cognitive function in mice	[202]
Resveratrol	APP; Aβ; microglia	CAT, SOD, NO, GSH; transition metal ions; ROS; PGC1-α	↓ Aβ aggregation in in the hippocampus and cortex of transgenic APP/PS1pa mice	[208, 209]
Ferulic acid	≠ BACE1 activity	SOD; LP; Drp1; Mfn2	↓ Aβ formation; maintains the functional state of mitochondria	[214, 215, 216]
Idebenone	ADAM17 and NEP; RAGE/ caspase-3	ROS	↓ Aβ deposition in 5xFAD mice; ↓ mitochondrial dysfunction	[217, 218]
α-lipoic acid	Aβ fibrils	ROS CAT, SOD, NO, GSH	↓ Aβ formation in vitro; ↓ OS	[219]
SS31	Aβ	Drp1 and Fis1; Mfn1/2 and OPA1; PGC1α and Nrf1/2	↓ Aβ formation; ↓ mitochondrial dysfunction; ↑ mitochondrial biogenesis	[220]
SkQ1	Aβ_1-40_ and Aβ_1-42_	Drp1 and Mfn2	↑ mitochondrial biogenesis; ↑ memory in OXYS rats; ↑ number of neurons in CA1 and CA3 areas and the dentate gyrus of OXYS rats; ↓ Aβ deposition	[221]

Note: ↓ – decreases; ↑ – increases; ≠ – inhibits.

NEP modulators [157], which facilitate Aβ clearance from the extracellular space, thus preventing Aβ entry into mitochondria and Aβ-induced mitochondrial dysfunction, are therapeutic targets in AD. Administration of the well-known antioxidant and HDAC inhibitor epigallocatechin-3-gallate (EGCG) reduces Aβ levels and increases NEP expression in the cerebral cortex of senescence accelerated (SAMP8) mice [158] and rats subjected to prenatal hypoxia [159]. In addition, EGCG suppresses BACE1 expression and decreases Aβ_1-42_ levels, improving learning and memory in a rat model of AD [160]. Li et al. found that (E)-N-((6-aminopyridin- 2-yl)methyl)-3-(4-hydroxy-3-methoxyphenyl)acrylamide inhibits BACE1 activity and exhibits strong antioxidant activity against 1,1-diphenyl- 2-picrylhydrazyl (DPPH) and 2,2’-azino-bis-(3-ethylbenzothiazoline-6-sulfonic acid) (ABTS), exceeding the effect of EGCG [161]. Another potential compound is Kai- Xin-San (KXS, a Chinese herbal decoction used to treat amnesia), which increases NEP levels in murine hippocampus [162]. A antioxidant activity of KXS was shown to exist in doxorubicin- [163] and scopolamine-induced models of OS [164]. KXS caused a simultaneous decrease in the malondialdehyde (MDA) level and increase in the activity of superoxide dismutase (SOD), glutathione peroxidase (GPx), and catalase (CAT). An antioxidant activity of KXS was also shown by Guo et al. [165].

A potential compound for the treatment of AD is the natural polyphenol curcumin, which has strong antioxidant activity [166, 167]. Curcumin neutralizes ROS, increases the levels of SOD, Na^+^-K+-ATPase, glutathione, and mitochondrial complex enzymes, and protects mitochondria from peroxynitrite [168, 169, 170, 171]. Another important property of curcumin is its ability to inhibit Aβ oligomerization and Aβ fibril formation, as well as hinder Aβ-induced neurotoxicity in the brain of transgenic mice [172]. Curcumin binds strongly to Aβ peptides through a wide range of intermolecular interactions: hydrogen bonds, hydrophobic interactions, π-π stacking, and cation-π-attraction. Curcumin forms π–π interactions with aromatic residues (Phe4, Tyr10, Phe19, and Phe20) and cation–π interactions with cationic residues (Arg5, Lys16, and Lys28) in Aβ [173]. Zhao et al. studied the effect of curcumin on the stability of Aβ dimers and found that curcumin disrupts β-sheets, reducing their number in Aβ oligomers [174]. In addition, curcumin binds strongly to the Aβ fibril pre-form, occupying a binding pocket inside the fibril, where it forms hydrogen bonds and hydrophobic interactions with protofibrils and causes structural distortions [175 , 176, 177]. In vivo and in vitro experiments revealed another mechanism of curcumin-induced reduction of Aβ accumulation and deposition: suppression of BACE1 expression [178, 179]. Hydroxylated derivatives of monocarbonyl curcumin containing cyclohexanone increase NEP levels [180]. Taken together, these data suggest that curcumin exhibits multi-targeted activity and warrants further study.

Another promising compound is the flavonoid silibinin (silybin), which has antioxidant activity [181]. Silibinin interacts with the mitochondrial membrane, preventing the dysfunction of isolated mitochondria [182]. Administration of silibinin decreases the MDA level and increases the activity of the antioxidant enzymes CAT, SOD, nitric oxide (NO), and glutathione (GSH) [183 , 184, 185, 186]. In addition to its antioxidant activity, silibinin can reduce Aβ deposition in the hippocampus of APP/PS1 mice by inhibiting APP and BACE1 expression and increasing the NEP level. The issue regarding the previously discovered inability of silibinin to pass through the BBB was solved by its encapsulation in macrophage-derived exosomes (Exo-Slb). After entering the brain of AD mice, Exo-Slb selectively interacts with Aβ monomers, preventing their aggregation, and effectively improves the memory of the animals [187]. The effect of silibinin encapsulated in the nanoparticles of human serum albumin (HSA) was also studied. The neuroprotective and antioxidant activity of silibinin–HSA nanoparticles was found to be higher than that of free silibinin [188]. Another flavonoid, quercetin, which modulates gene expression and the signaling pathways, also exhibits antioxidant and iron chelating activities [189]. Quercetin protects neurons from the action of H_2_O_2_ by reducing lactate dehydrogenase (LDH) release, ROS and MDA levels, while simultaneously increasing GPx and SOD activity [190]. Quercetin reduces mitochondrial dysfunction by reducing ROS production, restoring mitochondrial membrane production and ATP synthesis; it regulates the expression of AMPK, which is involved in the modulation of energy metabolism, reduces Aβ deposition, facilitates its excretion, and regulates APP processing [191]. Studies of transgenic AD mice have shown that quercetin decreases the level of extracellular Aβ [192, 193]. Oral administration of quercetin in rats with AlCl3-induced AD symptoms reduced Aβ aggregation in the hippocampus owing to a downregulation of APP, BACE1, APH1, and PSEN1 and overexpression of ADAM10 and ADAM17 [194]. The flavonoids taxifolin and isorhamnetin inhibit BACE1 activity and exhibit an antioxidant effect [195]. Taxifolin inhibits Aβ fibril formation in vitro and improves the cerebral blood flow, facilitating Aβ clearance [196]. Baicalein exhibits a number of important pharmacological properties as a neuroprotector: it reduces OS, inhibits Aβ aggregation, and stimulates neurogenesis [197]. Baicalein was also shown to prevent Aβ-induced neuronal atrophy and improve memory in mice [198]. The combination of baicalein and trans-chalcone significantly reduced the levels of ROS and Aβ_1-42_ in yeast cells expressing Aβ_1-42_ without affecting their growth in [199]. The neuroprotective mechanism of luteolin action consists in the direct inhibition of ROS and acetylcholinesterase (AChE) activity, as well as Aβ42 accumulation [200].

Numerous in vivo studies performed recently have shown the neuroprotective effect of the isoquinoline alkaloid berberine [201]. Berberine inhibits BACE1 and AChE activity, reduces the ROS level, while increasing the glutathione level, preventing apoptosis, and improving cognitive functions [202, 203]. The incorporation of berberine into lipid nano-carriers increased its bioavailability and effectiveness in an in vivo experiment [204]. It was also established that another natural alkaloid, piperine, and its metabolites can inhibit BACE1 and reduce the ROS level, thus decreasing the damage to mitochondria [205]. The sesquiterpene alkaloid huperzine A (HupA) also has a multifunctional activity: it reduces Aβ deposition in the cortex and hippocampus, improves mitochondrial functions, and inhibits AChE activity in an AD model of transgenic APPswe/PS1dE9 mice [206]. Recently, synthesized HupA analogues have demonstrated even higher efficiency [207].

Numerous studies have shown that polyphenol resveratrol exhibits a variety of biological activities, including antioxidant and neuroprotective effects. Resveratrol increases the expression and activity of antioxidant enzymes, binds transition metal ions, inactivates free radicals, and improves the mitochondrial function by increasing the expression and activation of the main inducer of mitochondrial biogenesis, PGC1-α [208]. Resveratrol reduces Aβ deposition through the activation of the non-amyloidogenic pathway of APP cleavage and Aβ excretion; it also activates microglia in the hippocampus and cortex of transgenic APP/PS1 mice [209]. Promising compounds exhibiting both antioxidant activity and the ability to inhibit BACE1 have been identified among derivatives of styryl benzamide [210], N-cyclohexylimidazo[1,2-a] pyridine [211], and trimethoxylated halogenated chalcones [212, 213].

The neuroprotective effect of ferulic acid (FA) can be implemented through several mechanisms. FA exhibits the antioxidant and mitoprotective effects. FA administration in a mouse model of AD increased SOD activity and decreased the MDA level [215]. In addition, FA restores the balance between mitochondrial fission and fusion by regulating the activity of fission and fusion proteins (by decreasing Drp1 expression and increasing Mfn2 expression) [216] and the PGC-1α level [222]. Maintenance of the PGC-1 level prevents a loss of the mitochondrial membrane potential and reduces Drp-1-dependent mitochondrial fission. The second important action of FA is its ability to inhibit BACE1, which prevents Aβ formation [214]. Promising compounds with anti-aggregation and antioxidant activities have also been identified among FA derivatives [223, 224].

Another direction in the search for AD drugs is the study of compounds that are similar to endogenous antioxidants. An example is idebenone, a coenzyme Q10 analogue that can pass through the BBB, which is an FDA-approved antioxidant. Idebenone inhibits Aβ-induced ROS production and mitochondrial dysfunction [217]. Idebenone administration significantly reduces Aβ deposition in 5xFAD mice by increasing the levels of α-secretase ADAM17 and NEP; it also inhibits the RAGE/caspase-3 signaling pathway [218]. The glutathione precursor N-acetylcysteine (NAC) reduced the levels of Aβ, phosphorylated tau, and OS markers and improved cognitive functions in animals in in vitro and in vivo experiments [225]. Alpha-lipoic acid (α-LA), whose production decreases with age, is considered a promising agent for the prevention and treatment of AD. This acid neutralizes ROS, increases the glutathione level, chelates transition metals, disrupts Aβ synthesis, and promotes its excretion [219]. In addition, α-LA acts as an enzyme cofactor capable of regulating the metabolism, energy production, and mitochondrial biogenesis [226]. The results of a randomized placebo-controlled trial showed that the combination of omega-3 fatty acid and α-LA delayed cognitive impairment in AD patients when administered for 12 months [227].

The antioxidant peptide SS31 reduces Aβ peptide production and restores mitochondrial and synaptic functions in a mouse model of AD [228]. The combined use of this peptide and mitochondrial division 1 inhibitor (Mdivi1) has a positive effect on cultured cells. This result suggests that combined treatment with the use of antioxidants acting on mitochondria may be more effective [229]. SkQ (10-(6’-plastoquinonyl) decylrhodamine 19), which accumulates mainly in neuronal mitochondria, improves the structural and functional state of organelles, thereby preventing neuronal loss and synaptic damage, and reduces the Aβ level and hyperphosphorylation of the tau protein in the hippocampus; this, in turn, leads to improved learning and memory ability in animals [221].

ABAD inhibitors are also promising agents in the search for anti-AD drugs. They prevent rapid binding of Aβ to ABAD in the mitochondrial matrix, resulting in PreP normalization [230, 231, 232, 233, 234].

Thus, the approach to the designing and developing of neuroprotective drugs based on combining various pharmacophore fragments in one molecule capable of acting on targets associated with proteinopathy and mitochondrial dysfunction is considered a promising and relevant strategy for medicinal chemistry and pharmacology.

## CONCLUSION

Due to the lack of effective drugs for the treatment of Alzheimer’s that have not only a symptomatic effect, but also a drastic impact on the disease’s pathological cascades, a targeted search for and development of drugs for a pharmacological correction of this neuronal disease remains relevant. In order to do this, it is necessary to understand not just individual pathogenetic processes, but their interrelation and how they mutually affect each other. For instance, the interaction between mitochondria and Aβ is a closely related process. Toxic forms of Aβ lead to mitochondrial dysfunction due to the impairment of Ca^2+^ homeostasis, mitochondrial fusion and fission, protein import, increased mitochondrial membrane permeability, and inhibition of mitochondrial respiratory chain complexes. At the same time, mitochondrial dysfunction leads to oxidative stress, energy crisis, and activation of cell death cascades. This, in turn, promotes processing of the precursor protein APP and leads to β-amyloid aggregation and deposition. Therefore, a more thorough understanding of the properties of potential neuroprotective drugs indicates that it is necessary to focus attention on the combination of pharmacophore fragments that can simultaneously affect the proteinopathy-associated cascades and prevent mitochondrial dysfunction in one molecule.

In this review, we tried to consolidate and analyze the currently available data on the role of Aβ interaction with mitochondria in the pathogenesis of Alzheimer’s disease and judge the effectiveness of the search for potential neuroprotective drugs targeting the pathological processes associated with proteinopathy and mitochondrial dysfunction.

## Abbreviations

AD: Alzheimer’s disease
Aβ: beta-amyloid peptide
APP: beta-amyloid precursor protein
MAM: mitochondria-associated endoplasmic reticulum membrane
ER: endoplasmic reticulum
TOM: translocase of the outer membrane
TIM: translocase of the inner membrane
BACE1: β-secretase 1
NEP: neprilysin, neutral endopeptidase
IDE: insulin-degrading enzyme
PreP: presequence protease (or pitrilysin metallopeptidase 1 (PITRM1))
ECE: endothelin-converting enzyme
ABAD: amyloid beta peptide-binding alcohol dehydrogenase
VDAC: voltage-dependent anion channel
PGC1α: peroxisome proliferator- activated receptor-γ coactivator 1-α
PINK1: PTEN-induced kinase 1
GSK3β: glycogen synthase kinase-3β
Fis1: mitochondrial fission protein 1
Drp1: dynamin-related protein 1
OPA1: optic atrophy type1
SOD: superoxide dismutase
GPx: glutathione peroxidase
CAT: catalase
GSH: glutathione
OS: oxidative stress
BBB: brain-blood barrier
LP: lipid peroxydation
